# The Inhibitory Mechanisms Study of 5,6,4′-Trihydroxy-7,3′-Dimethoxyflavone against the LPS-Induced Macrophage Inflammatory Responses through the Antioxidant Ability

**DOI:** 10.3390/molecules21020136

**Published:** 2016-01-22

**Authors:** Shih-Hao Wang, Chia-Hua Liang, Fong-Pin Liang, Hsiou-Yu Ding, Shiuan-Pey Lin, Guan-Jhong Huang, Wen-Chuan Lin, Shin-Hun Juang

**Affiliations:** 1 School of Pharmacy, China Medical University, Taichung 404, Taiwan; isaacwangsh@gmail.com (S.-H.W.); splin@mail.cmu.edu.tw (S.-P.L.); wclin@mail.cmu.edu.tw (W.-C.L.); 2 Department of Cosmetic Science, Chia Nan University of Pharmacy and Science, Tainan 717, Taiwan; tinna_ling@mail.cnu.edu.tw; 3 Department of Pharmacy, Tajen University, Pingtung 907, Taiwan; ultramarine@seed.net.tw; 4 Institute of Cosmetic Science, Chia Nan University of Pharmacy and Science, Tainan 717, Taiwan; hsiou221@yahoo.com.tw; 5 Department of Chinese Pharmaceutical Sciences and Chinese Medicine Resources, China Medical University, Taichung 404, Taiwan; gjhuang@mail.cmu.edu.tw

**Keywords:** *Anisomeles ovata*, 5,6,4′-trihydroxy-7,3′-dimethoxyflavone, anti-inflammatory, anti-oxidant, reactive oxygen species

## Abstract

The whole plant of *Anisomeles ovata* has been widely used in Taiwan for treating inflammation-related skin and liver diseases, however, the detailed pharmacology mechanisms have yet to be elucidated. In the present study, one of the major components, 5,6,4′-trihydroxy-7,3′-dimethoxyflavone (5-TDMF), was purified from a methanol extract of *Anisomeles ovata*. A pharmacological study of this compound suggests that 5-TDMF possesses potent free radical scavenging activity both *in vitro* and *ex vivo*. Furthermore, 5-TDMF reduces nitric oxide and pro-inflammatory cytokine production in LPC-treated RAW 264.7 cells through the attenuation of nitric oxide synthase and cyclooxygenase-2. Additional experiments suggest that of 5-TDMF interferes with nuclear factor-κB translocation and mitogen-activated protein kinase pathways. These results identify 5-TDMF as an anti-oxidant and anti-inflammatory compound, explain the pharmacologic function of *Anisomeles ovata* and suggest its great potential as a new anti-inflammatory remedy.

## 1. Introduction

Oxidative stress generally occurs when mitochondria overproduce reactive oxygen species (ROS) such as superoxide anion radicals (O_2_^•−^), hydrogen peroxide (H_2_O_2_), and hydroxyl radicals (OH^•^). The production of excess ROS molecules can lead to DNA, RNA, lipid, protein, and enzyme damage. Additionally, high levels of ROS production also leads to the activation of the NF-κB signaling pathway, up-regulation of inducible nitric oxide synthase (iNOS), cyclooxygenase-2 (COX-2) expression and induction of pro-inflammatory cytokines such as tumor necrosis factor-alpha (TNF-α) and interleukins [1,2,3]. Clinically, continuous exposures to oxidative damage have been correlated to cardiovascular diseases, diabetes, chronic inflammation and some types of cancer [4,5]. Therefore, identifying effective blocking agents of ROS production may serve as a potent anti-inflammation and cancer therapeutic.

*Anisomeles ovata* R. Br, also known as *Anisomeles indica* Kuntz., is the only species of the Labiatae family found in Taiwan and has been widely used as a herbal remedy to treat inflammation-related liver and skin diseases, hypertension and immune deficiencies [6]. Recent studies have suggested that *Anisomeles ovata* extracts exert various biological activities, including anti-oxidant, anti-inflammatory and tumor cell proliferation inhibitory [7,8,9,10], however, the underlying pharmacological mechanisms are still unclear. In order to identify the active ingredients of *Anisomeles ovata*, a number of chemical components have been identified, including flavonoids, terpenoids and steroids [9,11,12,13]. Among the isolated compounds, 5,6,4′-trihydroxy-7,3′-dimethoxyflavone (5-TDMF) was found to be the major flavonoid in the methanol extract of *Anisomeles ovata* [14]. However, a detailed pharmacological study of 5-TDMF has yet to be reported. In the present study, our results demonstrate that 5-TDMF shows potent free radical scavenging and anti-inflammatory activity without any observable cytotoxicity. Further experimental results also showed that 5-TDMF could block LPS-induced NF-κB translocation and iNOS and COX-2 expressions through inhibition of the mitogen-activated protein (MAP) kinase and Erk signaling pathways. Collectively, our study suggests that 5-TDMF may be a potent new chemopreventive anti-inflammatory agent.

## 2. Results

### 2.1. 5-TDMF Possesses Weak or no Cytotoxicity toward the Experimental Cell Lines

**Figure 1:**
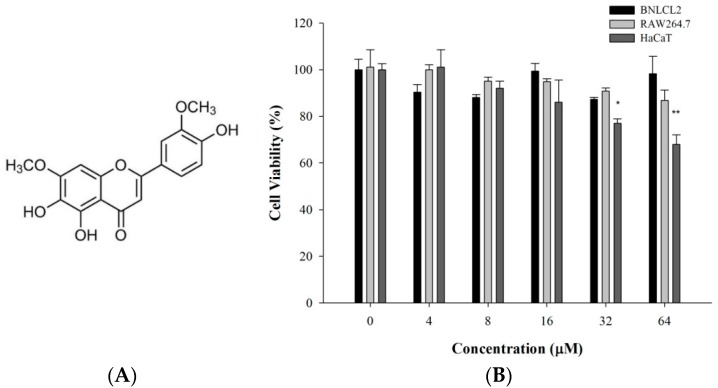
The cytotoxicity of 5-TDMF toward human keratinocytic cells (HaCaT), murine macrophage (RAW 264.7) and liver (BNLCL2) cells. (**A**) Chemical structure of 5-TDMF; (**B**) Cell viability as measured by an MTT assay after treatment with various concentrations of 5-TDMF for 72 h. The data were analyzed by Student’s t-test. Statistical significance is determined by * *p* < 0.05 and ** *p* < 0.01 relative to control.

To evaluate the cytotoxicity of 5-TDMF, HaCaT, BNLCL2 and RAW 264.7 cells were co-cultured with varying concentrations of 5-TDMF for 72 h and cell viability was determined by the MTT assay. Our results suggest that 5-TDMF is not cytotoxic to BNLCL2 and RAW 264.7 cells up to 64 μM. However, at higher concentrations of 5-TDMF, HaCaT proliferation was reduced by less than 30% (Figure 1B).

### 2.2. 5-TDMF Demonstrates Potent Inhibitory Activity of Lipid Peroxidation

**Figure 2:**
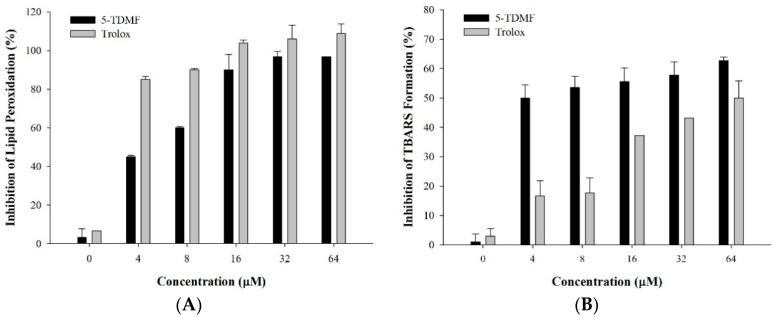
Inhibition of LP formation by 5-TDMF in (**A**) the liposome model and (**B**) mouse liver homogenate model. Varying concentrations of 5-TDMF and Trolox were added into the liposome suspension or mouse liver homogenates using the Fe^2+^/ascorbate system and the formation of LP was then measured as described in the materials and method section.

Lipid peroxidation (LP) is considered as the main molecular mechanism involved in the oxidative damage of cellular mitochondria and microsomes. In the presence of OH^•^, lipid peroxyl radicals (LOO^•^) are generated through the reaction of polyunsaturated fatty acids with oxygen. After cells are insulted by LOO^•^, thiobarbituric acid reactive substances (TBARS) can be generated from the breakdown of peroxides and can be used to monitor cellular damage [15]. In this study, the ability of 5-TDMF to protect against LP was evaluated by the degree of reduction of TBARS using liposome and mouse liver homogenate models [16,17]. In the liposome model, our results indicate that the inhibition of LP by 5-TDMF was concentration-dependent, with an EC_50_ of 4.5 µM. While low concentrations of 5-TDMF demonstrated weaker LP protection compared to Trolox, at concentrations of 16 µM and higher, 5-TDMF displayed comparable LP protective properties as Trolox (Figure 2A). Furthermore, the inhibition of TBARS production in the mouse liver homogenate model by 5-TDMF also showed a concentration-dependent relationship. The inhibitory effects of 5-TDMF reached as high as 60% when liver cells were co-cultured with 32 µM 5-TDMF (Figure 2B). Collectively, our results demonstrate that 5-TDMF possesses similar or better LP inhibitory activity compared to Trolox.

### 2.3. 5-TDMF is a Potent Free Radical Scavenger and Anti-Oxidant

**Figure 3:**
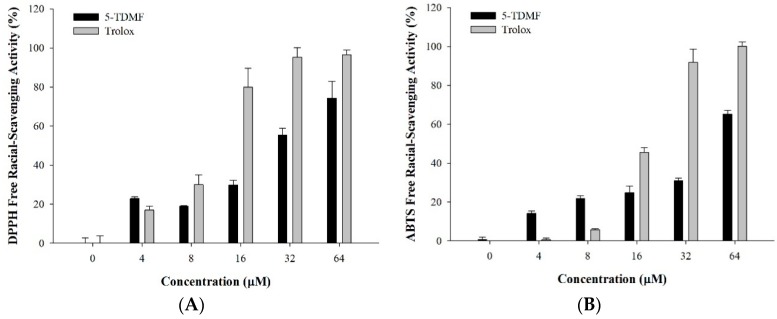

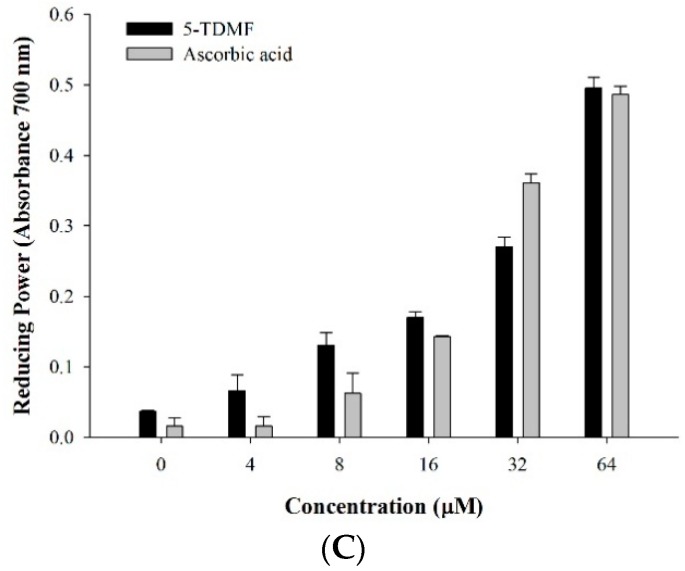
Evaluation of anti-oxidant activity of 5-TDMF *in vitro*. Varying concentrations of 5-TDMF and Trolox were added into the reaction buffer of DPPH^•^ (**A**) and ABTS^•+^ (**B**) and the formation of DPPH^•^ and ABTS^•+^ was measured; (**C**) Reducing power of 5-TDMF and ascorbic acid by the Oyaizu method.

In order to examine the LP protective mechanisms of 5-TDMF, several experiments to measure free radical scavenging capabilities were performed. First, the 5-TDMF reductive ability towards two potent radical molecules, DPPH^•^ and ABTS^•+^ were measured. Our results showed that 5-TDMF exhibited potent free radical scavenging activity in a concentration-dependent manner with an EC_50_ value of 26.9 µM for DPPH^•^ and 47.2 µM for ABTS^•+^ (Figure 3A,B), and accordingly, the Trolox equivalent anti-oxidant capacity (TEAC) value derived from the dose-response curve for 5-TDMF was 1.4 mM Trolox/g.

Additionally, a compound’s reducing capabilities can be used as an indicator of its potential as an anti-oxidant [5]. Accordingly, the reducing power of 5-TDMF was determined by the Oyaizu method [18]. The results showed that 5-TDMF exhibited significant activity against Fe^2+^ formation. At low concentrations, 5-TDMF and ascorbic acid have comparable reducing power. However, at mid-range concentrations, 8 µM of 5-TDMF was found to be as efficient as 16 µM of ascorbic acid (Figure 3C).

### 2.4. 5-TDMF Showed Potent Cellular Radical-Scavenging Activity by Suppressing the Intracellular ROS Formation and Up-Regulating GSH Expression

**Figure 4:**
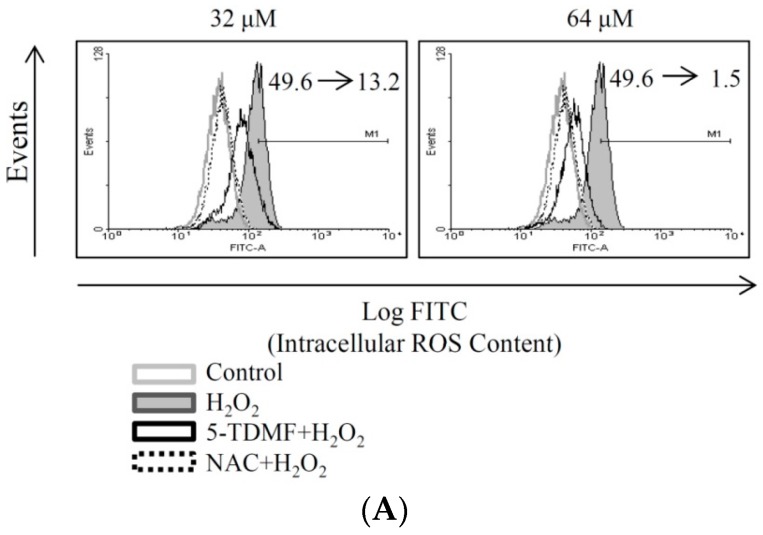

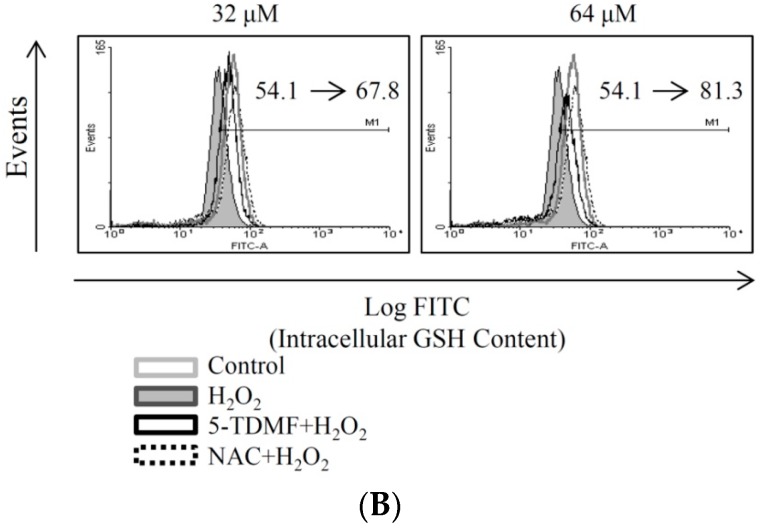
Evaluation of anti-oxidant activity of 5-TDMF *ex vivo*. Cells were pretreated with 32 or 64 μM 5-TDMF for 24 h and H_2_O_2_ (0.1 mM) was added for 1 h. The H_2_O_2_-treated cells were collected and ROS (**A**) and GSH (**B**) levels were measured as describe in the materials and method section.

Examining ROS and GSH levels is widely accepted as a measurement of oxidative stress and can be used to monitor the effectiveness of an anti-oxidant intervention strategy. Therefore, the ROS and GSH levels of H_2_O_2_-treated BNLCL2 cells in the presence or absence of 5-TDMF was determined. The results showed that pretreated with 5-TDMF can significantly reduce the levels of ROS in H_2_O_2_-treated BNLCL2 cells with the geometric mean value reduced from 49.6 to 13.2 and 1.5 when cells were pretreated with 32 and 64 μM of 5-TDMF, respectively (Figure 4A). Furthermore, our results showed that significant amount of GSH can be resorted, the geometric mean was restored from 54.1 to 67.78 and 81.25, by pre-treatment of 5-TDMF of 32 and 64 μM of 5-TDMF respectively (Figure 4B). NAC, the positive control, reduced the levels of ROS from 49.6 to 0.06 and restored the levels of GSH from 54.1 to 86.

These results suggested that 5-TDMF can inhibit ROS formation and GSH expression in cells exposed to H_2_O_2_. Hence, these results suggest that the stronger radical-scavenging capacity of 5-TDMF acts as both primary and secondary anti-oxidants by inhibiting the formation of lipid peroxyl radicals.

### 2.5. 5-TDMF Reduced LPS-Induced Inflammatory Responses in RAW 264.7 Cells

**Figure 5:**
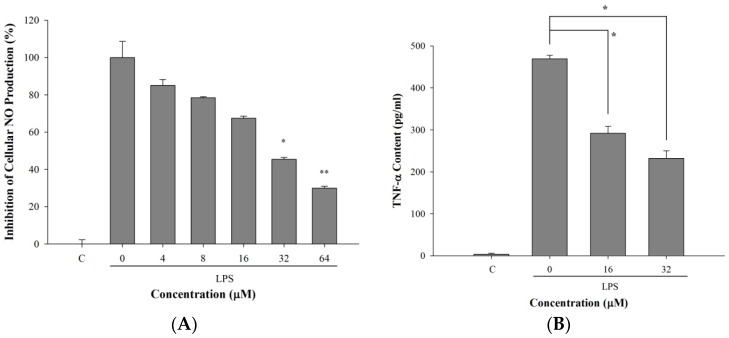
The anti-inflammatory effects of 5-TDMF towards the LPS-treated RAW 264.7 cells. RAW 264.7 cells were pre-treated with or without 5-TDMF for 12 h. After stimulation with LPS (100 ng/mL), RAW 264.7 cells were co-cultured in the presence or absence of 5-TDMF for 24 h and the inhibitory effects of 5-TDMF on NO (**A**) and TNF-α (**B**) production were measured. ANOVA follows Dunnett’s test. Statistical significance is determined by * *p* < 0.05 and ** *p* < 0.01 relative to LPS.

LPS stimulation induces inflammatory responses from macrophages resulting in NO and TNF-α up-regulation [19,20]. Since *Anisomeles ovata* has been widely used as an anti-inflammatory herb, we wanted to examine the anti-inflammatory potential of 5-TDMF. The ability of 5-TDMF to inhibit LPS-induced NO production in RAW 264.7 cells was measured by the Griess reagent system [21]. Results showed that NO production in RAW 264.7 cells was increased by LPS treatment, however, pre-treatment with 5-TDMF significantly suppressed LPS-induced NO production with an EC_50_ value of 9.9 μM (Figure 5A). Since previous results showed that 5-TDMF presented no significant cellular toxicity toward RAW 264.7 cells up to 64 μM, the down-regulation of NO production by 5-TDMF in LPS-treated RAW 264.7 cells was not due to cell death.

Furthermore, the anti-inflammatory activity of 5-TDMF was evaluated toward the LPS-treated RAW264.7 cells. Our results showed that pre-treatment of 5-TDMF suppressed LPS-induced production of TNF-α in RAW 264.7 cells. Compared to the untreated group, the amount of TNF-αfound in 5-TDMF-treated (32 μM) RAW 264.7 cells was down-regulated by 50% (Figure 5B). The above results suggested that 5-TDMF can reduce LPS-induced NO and TNF-αproduction in macrophage cells.

### 2.6. Pre-Treatment with 5-TDMF Can Suppress LPS-Induced NF-κB and MAP Signaling Pathway Resulting in the Down-Regulation of iNOS and COX-2 Expression

**Figure 6:**
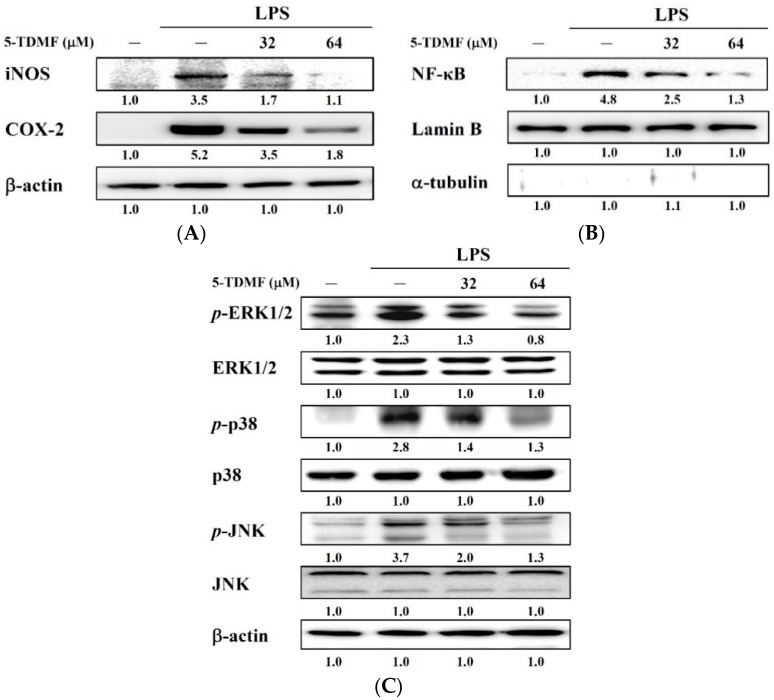
5-TDMF inhibits LPS-induced inflammatory responses. The RAW 264.7 cells were pre-treated with or without 5-TDMF for 12 h and then LPS (100 ng/mL) for 30 min. iNOS and COX-2 (**A**); nuclear NF-κB (**B**) and ERK1/2, p38 and JNK (**C**) expression were examined by western blot analysis.

In order to delineate the molecular mechanisms of 5-TDMF mediated down-regulation of NO and TNF-α, protein expression levels of two upstream signal proteins iNOS and COX-2, were investigated. Our results showed that LPS-induced iNOS and COX-2 protein in RAW 264.7 cells can be suppressed by pre-treatment with 5-TDMF in a concentration-dependent manner (Figure 6A).

Previous studies showed that free radical could up-regulation iNOS and COX-2 expression through activation of NF-κB nuclear translocation [22,23], therefore whether 5-TDMF could interfere NF-κB p65 translocation was investigated. The Western blots results demonstrated that pre-treatment with 5-TDMF can significantly decrease LPS-induced NF-κB p65 protein nuclear translocation (Figure 6B) without changing the total NF-κB p65 protein expression levels (data not showed). Furthermore, the level of *p*-ERK1/2, *p*-p38 and *p*-JNK was significantly suppressed in a 5-TDMF concentration-dependent manner (Figure 6C). These results indicate that 5-TDMF suppressed expression of iNOS and COX-2 through inhibition of NF-κB and the down-regulation of the MAP and Erk signaling kinases.

## 3. Discussion

Oxidative stress typically occurs because of an imbalance between the production of reactive oxygen species (ROS) and anti-oxidants [24]. Oxidative stress can disrupt the DNA and RNA stability, perturb proteins function and induce lipid peroxidation then cause serious cellular damage.

Lipid peroxidation can be described generally as a process whereby oxygen insertion into polyunsaturated fatty acids after attack by free radicals results in lipid peroxyl radicals and hydroperoxides [25]. These highly reactive electrophiles readily react with cellular proteins and nucleic acids initiating signaling cascades and activating transcription factors resulting in inflammation [26].

Recent studies have shown that inflammation can up-regulate many pro-inflammatory mediators such as iNOS and COX-2 [1,27] and these two enzymes are also activated by the key transcription factor NF-κB resulting in stress responses, inflammation, and apoptosis [28]. In addition, the induction of NF-κB activity can also be modulated by the MAPK family, including Erk1/2, p38 MAPK and JNK which are known to be involved in various cellular functions [1,29]. Based on these observations, identifying an effective agent to block ROS production may serve as a rational approach to developing novel anti-inflammatory treatments.

The extract of *Anisomeles ovata* has long been used for the treatment of diverse inflammatory relative skin and liver diseases in Taiwan [9], however, the active ingredients and the molecular mechanisms of the anti-inflammatory activity remained elusive. In the present study, one of the major components of methanol-extracted *Anisomeles ovata*, 5-TDMF, was isolated and its pharmacological activity evaluated for free radical-scavenging and anti-inflammatory potential.

Our results indicate that 5-TDMF shows very weak cytotoxicity (Figure 1B) but possesses potent inhibitory effects against Fe^3+^-induced lipid peroxidation and TBARS production (Figure 2A,B). Moreover, a concentration-dependent anti-oxidant activity of 5-TDMF was found against DPPH^•^ and ABTS^•+^ with the EC_50_ values of 26.9 μM and 47.2 μM, respectively (Table 1) and it showed better reducing power than an equivalent concentration of ascorbic acid (Figure 2C). Furthermore, the protective action of 5-TDMF against H_2_O_2_-induced BNLCL2 cell damage was demonstrated through the reduction of H_2_O_2_-induced ROS production and restored the H_2_O_2_-suppressed GSH expression (Figure 4A,B). Collectively, our results clearly demonstrate the potential multiple anti-oxidative functions of 5-TDMF.

**Table 1:** The effective concentrations of 5-TDMF *in vitro* antioxidant assays.

Sample	Lipid Peroxidation	TBARS Levels	DPPH^•^	ABTS^•+^
	EC_50_ (μM)	EC_50_ (μM)	EC_50_ (μM)	EC_50_ (μM)
5-TDMF	4.5	3.0	26.9	47.2
Trolox	2.4	80.0	14	21.5

Previous studies showed that ROS could alter the activity of ERKs, p38 and JNKs, and transcription factors which involved in inflammation [3,30]. Therefore, whether the down-regulation of ROS content of 5-TDMF-treatment might be involved in anti-inflammation was investigated. Our results clearly showed that 5-TDMF-treatment reduced LPS-induced NO and TNF-α expression in RAW 264.7 cells (Figure 5A,B). Furthermore, pre-treatment with 5-TDMF inhibited the LPS-induced MAPK/ERK signaling pathway (Figure 6C), suppressed the LPS-induced NF-κB nuclear translocation (Figure 6B) resulting in down-regulation of iNOS and COX-2 expression (Figure 6A).

**Figure 7:**
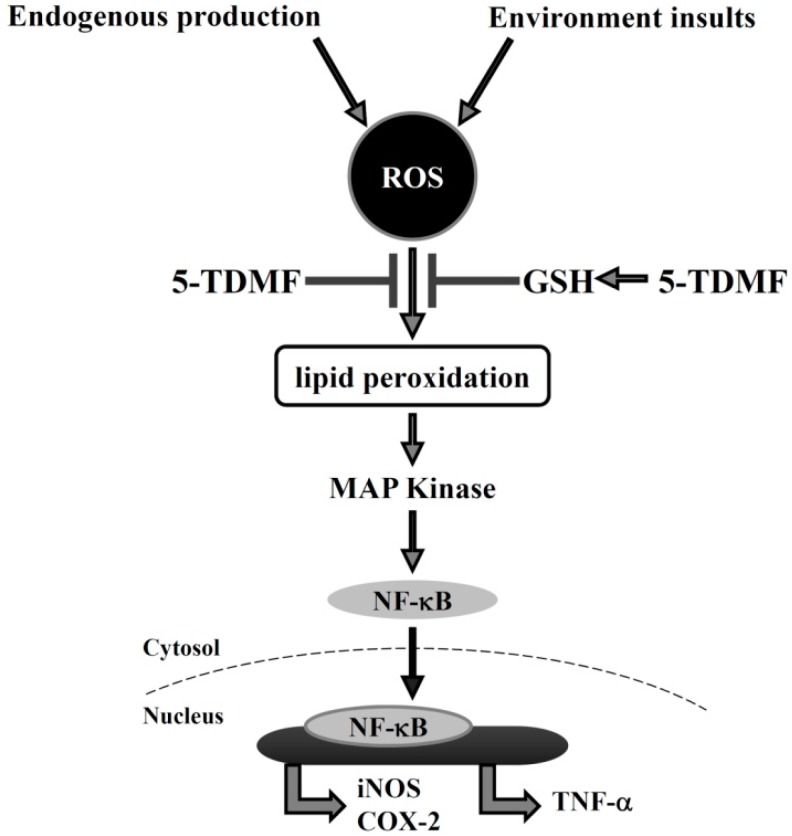
Possible molecular mechanisms underlying the anti-inflammatory activities of 5-TDMF.

In conclusion, our findings suggest that 5-TDMF possesses potent reducing ability, free radical scavenge activity and an ability to restore GSH expression under the ROS environment. The reduction of cellular ROS accumulation following 5-TDMF treatment could protect lipid peroxidation and reduce LPS-activated MAP kinase and NF-κB signaling pathways. The reduction of LPS-induced NF-κB activation could further block ROS-induced inflammation respond (Figure 7). Our results provide strong mechanistic evidence to explain the anti-inflammatory properties of *Anisomeles ovata*, in particular, 5-TDMF as a potent anti-inflammatory compound worthy of further development. The pharmacological activities of additional *Anisomeles ovata* extracts are also active investigations in our laboratory.

## 4. Materials and Methods

### 4.1. Isolation of 5-TDMF from the Whole Plant of Anisomeles Ovata

The 5-TDMF used in this study was purified as a yellow power from the methanol-fraction of *Anisomeles ovata* by our team following the published procedure [14]. The purified 5-TDMF exhibited similar spectroscopic data (IR, NMR, MS) to a previous publication [31]. In the study, 5-TDMF was dissolved in dimethyl sulfoxide (DMSO) and the final DMSO concentration did not exceed 0.1%.

### 4.2. Cell Culture

Human keratinocytic cells HaCaT were kindly provided by Prof. Hamm-Ming Sheu (National Cheng Kung University Medical College, Tainan, Taiwan). Murine normal embryonic liver BNLCL2 and Murine monocyte-macrophage RAW 264.7 cells were purchased from the Bioresource Collection and Research Center (BCRC 60180 and BCRC 60001, Hsinchu, Taiwan). Cells were cultured in Dulbecco’s Modified Eagle Medium (DMEM) medium supplemented with 10% fetal bovine serum at 37 °C in a humidified atmosphere of 5% CO_2_ in the presence of penicillin/streptomycin and l-glutamine.

### 4.3. Chemicals and Reagents

1,1-Diphenyl-2-picrylhydrazyl (DPPH^•^), 2,2′-azinobis (3-ethylbenzothiazoline-6-sulphonic acid) diammonium salt (ABTS^•+^), 2,5,7,8-tetramethylchromancarboxylic acid (Trolox), 2-thiobarbituric acid (TBA), ascorbic acid, lipopolysaccharide (LPS), *N*-(1-naphthyl)ethylenediamine, sulfanilamide, ethylene diaminetetraacetic acid disodium salt dehydrate (EDTA), hydrogen peroxide (H_2_O_2_), trichloracetic acid (TCA), *N*-acetyl-l-cysteine (NAC) and Trolox were purchased from Sigma Chemical Co. (St. Louis, MO, USA). The 3-(2-pyridyl)-5,6-diphenyl- 1,2,4-triazine-4′,4′′-disulphonic acid monosodium salt (ferrozine) was purchased from Fluka (Buchs, Switzerland). All the chemicals were analytical grade or higher. Primary antibody to α-tubulin was purchased from Sigma-Aldrich LLC; antibodies against COX-2, iNOS, *p*-ERK, *p*-JNK, *p*-p38, lamin B, NFκB p65 and β-actin, as well as horseradish peroxidase-conjugated secondary antibody were purchased from Santa Cruz Biotechnology (Santa Cruz, CA, USA).

### 4.4. Assessment of Cell Viability

The colorimetric assay for cellular growth and survival was performed as described by Hansen *et al.* with modifications (Hansen, Nielsen, and Berg, [32]). Briefly, HaCaT, BNLCL2 and RAW 264.7 cells (1.5 × 10^4^ cells/well) were seeded in 96-well plates overnight and various concentrations of 5-TDMF was added for an additional 72 h. Two hours before the end of the incubation, 3-(4,5-cimethylthiazol-2-yl)-2,5-diphenyltetrazolium bromide (MTT) was added to a final concentration of 5 μg/mL. Afterwards, solubilization buffer (40% DMF and 20% SDS in H_2_O) was added to wells to dissolve violet formazan precipitation overnight at 37 °C. The absorbance at 570 nm was then detected by a microplate reader and the IC_50_ value was calculated by linear regression analysis.

### 4.5. Free radical Scavenging Activity

The free radical scavenging activity of 5-TDMF toward DPPH^•^ and ABTS^•+^ was measured as previously published [33,34]. Various concentrations of 5-TDMF or Trolox were added to the DPPH^•^ (0.1 mM) or ABTS^•+^ solution and incubation conditions were performed as previously published [5]. For the DPPH^•^ assay, the absorbance (A) at 517 nm was measured after a 30 min incubation in the dark. For the ABTS^•+^ assay, the absorbance (A) at 734 nm was measured after a 30 min incubation in the dark at 30 °C. Trolox was taken as standard antioxidants. Free radical scavenging activity was calculated based on the following formula: (%) = (1) A_sample_/A_control_) × 100. The DPPH^•^ and ABTS^•+^ radical scavenging activities were calculated while EC_50_ values were estimated using a linear regression algorithm. The material concentration showing the same percentage change of absorbance of the ABTS^•+^ as that of 1 mM Trolox was defined as Trolox equivalent anti-oxidant capacity (TEAC). The data are presented as mean values ± standard deviation (*n* = 3).

### 4.6. Reducing Power

The Oyaizu method [18] was used to determine the reducing power of 5-TDMF. Briefly, various concentrations of 5-TDMF or ascorbic acid (0.2 mL) were added to a mixture of sodium phosphate buffer/potassium ferricyanide (K_3_Fe(CN)_6_) solution and incubated at 50 °C for 20 min. The reaction was stop by adding 0.5 mL 10% TCA (10%, *w*/*v*) then subjected to centrifugation at 10,000 *g* for 10 min. The supernatant was then mixed with ferric chloride (FeCl_3_) (0.1%, *w*/*v*) solution at room temperature. Ascorbic acid was taken as standard antioxidants. The reducing power was measured as the formation of complexes with Fe^2+^ at an absorbance of 700 nm.

### 4.7. Inhibition of Lipid Peroxidation Formation

Lipid peroxidation was determined using the liposome model as biological membranes as previously described [33,34]. As a model system of biological membranes, the commercial preparation of liposomes, pH 5–7, was used. The liposomes, 225–250 nm in diameter, were obtained by dissolving the commercial preparation in demineralized water (1:10) in an ultrasonic bath. For measuring liposome peroxidation formation, 32 μL liposome suspensions were incubated with 11 μL of 10 mM FeSO_4_, 11 μL of 10 mM ascorbic acid in the presence or absence of various concentration of 5-TDMF or Trolox in 1.515 mL of 50 mM Na_2_HPO_4_-NaH_2_PO_4_ buffer, pH 7.4 (2.5 mL final solution) for 1 h at 37 °C. The reaction was terminated by adding a cocktail of 0.8 mL of 1% 2-thiobarbituric acid and 10% trichloroacetic acid reagent and 106 μL of 0.1 M EDTA at 100 °C for 20 min. After cooling and centrifugation (2600 *g* for 10 min), malonaldehyde formation was measured at 532 nm. Trolox was taken as standard antioxidants. The percentage of inhibition of lipid peroxidation = (1 − A_sample_/A_control_) × 100 was calculated, and EC_50_ values were determined. The data are presented as mean values ± standard deviation (*n* = 3).

### 4.8. Inhibition of Thiobarbituric Acid-Reactive Substances Formation

Lipid peroxides formation of liver tissues was measured as the formation of TBARS following the Chou’s method [17]. Seven-week-old male Institute of Cancer Research (ICR) mice (National Animal Laboratory Center, Taipei, Taiwan) weighing 30–35 g were used. Tissue lipid peroxides produced were monitored by measuring concentrations of TBARS. In brief, liver tissues were homogenized with a Polytron in ice-cold Tris-HCl buffer (40 mM, pH 7.4) to produce a 1:1 (*w*/*v*) tissue homogenate, which was centrifuged at 3000 *g* for 10 min. 100 μL of supernatant was incubated with various concentration of 5-TDMF or Trolox (200 μL) in the presence of FeSO_4_ (10 μM, 100 μL) and ascorbic acid (0.1 mM, 100 μL) at 37 °C for 1 h. The reaction was terminated by the addition of TCA (28% *w*/*v*, 500 μL), followed by TBA (1% *w*/*v*, 380 μL), and the mixture was then heated at 80 °C for 20 min. After centrifugation at 3000 *g* for 10 min, the TBARS that formed in the supernatant was measured by absorbance at 532 nm. Trolox was taken as standard antioxidants. The inhibition ratio (%) was calculated by using the following formula: inhibition ratio (%) = (1 − A_sample_/A_control_) × 100.

### 4.9. Measurement of Reactive Oxygen Species and Glutathione Production

The ROS inhibitory activity of 5-TDMF was measured as described by Bass [35]. The BNLCL2 Cells (1 × 10^5^ cells/mL) were co-cultured with or without 5-TDMF (32 and 64 μM) for 24 h. NAC was taken as positive control. After incubation, 0.1 mM H_2_O_2_ was added and the mixture was incubated at 37 °C for 1 h then culture medium was replaced with fresh medium containing 10 μM DCFH-DA. After 30 min of incubation at 37 °C, fluorescence intensities of DCF were measured on a Canto II flow cytometer (BD Biosciences, San Jose, CA, USA). To measure the 5-TDMF-induced GSH levels, the o-phthalaldehyde (OPA) conversion method by Senft was used [36]. 50 μg/mL of OPA in 0.1 M Na_2_HPO_4_-5 mM EDTA buffer was added to the 5-TDMF-treated cells and the reaction was kept in the dark at 25 °C for 1 h. After two wash steps with phosphate buffer (pH 7.0), the intensity of GSH fluorescence was analyzed on a Canto II flow cytometer (BD Biosciences).

### 4.10. Measurements of LPS-Induced NO Production and Inflammatory Cytokines Production

The Griess reaction [21] was used to measure the production of NO. RAW 264.7 cells were seeded at a density of 1 × 10^4^ cells/well in 96 well-plates. After 24 h, cells were pretreated with various concentrations of 5-TDMF for 12 h and then 100 ng/mL LPS was added for another 24 h. At the end of incubation, 0.1 mL of supernatant from each sample was incubated with the same volume of Griess reagent and the NO production was measured at 540 nm. The inhibition of NO production was calculated using the following inhibition rate formula: (%) = (1 A_sample_/A_control_) × 100 and the results were expressed as IC_50_ values, which were defined as the concentration that inhibit 50% of NO production.

To measure the amounts of LPS-induced TNF-α in the cell culture supernatant, Mouse TNF (Mono/Mono) ELISA Set from BD Biosciences were used following the manufacturer’s instructions.

### 4.11. Preparation of Cell Lysates and Western Blot Analysis

RAW 264.7 cells were seeded at 5 × 10^5^ cells in a 10 cm plates overnight and pretreated with 32 μM of 5-TDMF for 12 h. A final concentration of 100 ng/mL of LPS was added into 5-TDMF-pre-treated cells for a pre-determined period. To collect the total cellular protein, cells were collected, lysed in ice-cold lysis buffer (100 mM Tris (pH 7.4), 1% NP40, 0.01% SDS, 1 mM PMSF, 10 µg/mL pepstatin, and 30 µg/mL leupeptin), and subjected to centrifugation to remove the insoluble material. To analysis the level of nuclear NF-κB, the nuclear and cytoplasmic proteins were separated by a NE-PER™ Nuclear and Cytoplasmic Extraction Reagents kit (Thermo Scientific™, Waltham, MA, USA).

Protein concentrations of lysates were determined using the BCA Protein Assay Reagent (Pierce Biotechnology, Rockford, IL, USA). Equal amounts of protein from each sample was separated on a SDS-PAGE gels and transferred to PVDF-membranes. After soaking in a blocking solution consisting of TBS with 0.05% Tween 20, and 5% skim milk, the blot was incubated with the primary antibody and antibody binding was detected using the appropriate secondary antibody coupled with horseradish peroxidase according to the manufacturer’s instructions. Enhanced chemiluminescence was used to detect the relevant proteins following protocols suggested by the manufacturer and then images were taken on a LAS-4000 (Fuji Film, Tokyo, Japan).

### 4.12. Statistical Analysis

Each experiment was repeated at least three times and the data was expressed as means with standard deviations (SD). Statistical differences were estimated by one-way analysis of variance (ANOVA) following Dunnett’s test. *p*-Value less than 0.05 was considered as statistically significant.
